# The Role of Nucleotide Excision Repair in Cisplatin-Induced Peripheral Neuropathy: Mechanism, Prevention, and Treatment

**DOI:** 10.3390/ijms22041975

**Published:** 2021-02-17

**Authors:** Scarlett Acklin, Fen Xia

**Affiliations:** Department of Radiation Oncology, Rockefeller Cancer Institute, University of Arkansas for Medical Sciences, Little Rock, AR 72205, USA; smacklin@uams.edu

**Keywords:** nucleotide excision repair, cisplatin, peripheral neuropathy, chemotherapy-induced peripheral neuropathy

## Abstract

Platinum-based chemotherapy-induced peripheral neuropathy (CIPN) is one of the most common dose-limiting effects of cancer treatment and results in dose reduction and discontinuation of life-saving chemotherapy. Its debilitating effects are often permanent and lead to lifelong impairment of quality of life in cancer patients. While the mechanisms underlying the toxicity are not yet fully defined, dorsal root ganglia sensory neurons play an integral role in symptom development. DNA-platinum adducts accumulate in these cells and inhibit normal cellular function. Nucleotide excision repair (NER) is integral to the repair of platinum adducts, and proteins involved in its mechanism serve as potential targets for future therapeutics. This review aims to highlight NER’s role in cisplatin-induced peripheral neuropathy, summarize current clinical approaches to the toxicity, and discuss future perspectives for the prevention and treatment of CIPN.

## 1. Chemotherapy-Induced Peripheral Neuropathy

Taxanes (i.e., paclitaxel, docetaxel), proteasome/angiogenesis inhibitors (bortezomib/thalidomide), vinca alkaloids (i.e., vincristine, vinorelbine), and platinum-based drugs (i.e., cisplatin, oxaliplatin) are the most common systemic anticancer treatments used as first-line chemotherapy for a variety of cancers, including breast, lung, colorectal and gastric cancers, and multiple myeloma [1]. While systemic chemotherapeutics offer potentially curative effects for cancer patients, they also confer a variety of neurotoxicities which can lead to dose reductions. In extreme cases, it can lead to cessation of chemotherapy treatment entirely [2]. Particularly, platinum-based chemotherapeutic agents, such as cisplatin, are well known to cause systemic neuronal toxicity leading to diffuse, bilateral degenerative changes in peripheral sensation and an altered perception of cold, heat, and pain. Clinically, chemotherapy-induced peripheral neuropathy (CIPN) is typically characterized by a subacute development of numbness, paresthesia, and occasional pain. Symptoms usually follow a “stocking and glove” distribution, first affecting the fingers and toes before migrating proximally to involve the arms and legs, respectively. Decreased sensitivity to vibration in the toes and loss of ankle jerk reflexes are the first observable signs of cisplatin-induced peripheral neuropathy. Despite loss of reflexes and proprioception impairment, pinprick, temperature sensation, and motor strength are initially maintained or less severely affected. Autonomic neuropathy was previously reported in multiple case reports; however, neuropathy rarely affects the autonomic nervous system. With prolonged treatment, patients develop significant burning, shooting, or electric-shock-like pain in the same distribution [3,4,5]. Signs also include generalized loss of deep tendon reflexes and worsening proximal impairment in vibration sensation. Rarely, patients may develop Lhermitte’s sign—an electric-shock-like sensation radiating cervico-caudally along the spine that can involve the arms, legs, or both and is provoked by neck flexion or rotation. It has been described in patients with direct tumor involvement of the spinal cord, in relation to radiotherapy, and with cisplatin treatment, and is believed to result from a transient demyelination of the posterior columns [6]. These painful symptoms of CIPN may persist well beyond discontinuation of treatment, sometimes permanently, and ultimately impede the quality of life of cancer patients. Prevalence of CIPN is also high. Rates have been reported as high as 84% in patients receiving cisplatin treatments [7]. Importantly, the likelihood of developing cisplatin-induced peripheral neuropathy is dose- and duration-dependent. Onset of the toxicity is expected to occur following cisplatin treatment at 250–350 mg/m^2^, and cumulative doses of 500–600 mg/m^2^ result in development of CIPN in almost all patients [8]. As cancer prevalence continues to increase, and along with it the use of chemotherapy, CIPN has become an urgent, unresolved medical problem for which there are no effective treatments or preventive measures available [3,9,10,11].

## 2. Platinum-Based Chemotherapy Agents: Mechanism of Action in Cancer Control and CIPN

Since cisplatin gained approval by the US Food and Drug Administration in the late 1970s for the treatment of testicular, ovarian, and bladder cancer, additional platinum-based chemotherapeutics have been developed, and clinical indications have grown to include several cancer types [12]. In fact, platinum-based drugs continue to be some of the mostly widely used anticancer treatments. However, despite over 40 years of research and identification of DNA as the major cellular target early on, the mechanisms involved in platinum-based therapy and related toxicity remain to be fully elucidated. Toxicity profiles and dose-limiting side effects differ between platinum drugs. Of the three agents currently used in the United States—cisplatin, carboplatin, and oxaliplatin—cisplatin and oxaliplatin have a higher neurotoxic potential than carboplatin. This difference in neurotoxicity reflects the reactivity of a specific platinum drug, specifically the lability of leaving groups as they bind different biomolecules, and determines the severity of the toxicity. Consequently, more labile drugs are more toxic. This lability is conferred by the positive charge created on metal ions with vacant d-orbitals which allows the ions to bind electronegative sites on proteins and nucleic acids [13]. All three agents inhibit normal DNA function through the formation of monoadducts and DNA crosslinks, processes that are exploited in anticancer therapy due to inherent differences between healthy tissue and cancer cells. Much of tumors’ sensitivity to platinum-based chemotherapeutics comes from differences in the DNA damage response (DDR) [14,15], as the majority of cancers are defective in at least one DDR pathway [16]. Of the four major repair pathways—double-strand break (DSB) repair, base excision repair, nucleotide excision repair (NER), and mismatch repair—NER is particularly relevant to platinum agents as discussed below.

Multiple mechanisms underlying neurotoxicity resulting from platinum-based chemotherapy have been proposed, yet treatment modalities remain elusive. Bodies of sensory neurons within the dorsal root ganglia (DRG) are believed to be the primary target of platinum agents [17,18,19], although Schwann cells, Langerhans cells, and macrophages could also play a role [20,21,22]. Increasing evidence shows hypersensitivity to mechanical and thermal stimuli commonly develops after preferential damage to DRG sensory fibers. This may be particularly relevant to CIPN due, in part, to the lack of the blood-brain barrier in the peripheral nervous system and the consequent exposure of its neurons to endogenous and exogenous agents, such as metabolites, inflammatory molecules, and environmental contaminants. Moreover, cisplatin has been demonstrated to preferentially bind to DNA in DRG neurons with a high propensity for platinum adduct formation. In addition to DNA injury, oxidative stress, and mitochondrial dysfunction [23,24,25], dysregulation of intracellular signaling pathways [26,27,28], voltage-gated ion channel dysfunction [29,30,31], and neuroinflammation [32] are among the proposed underlying mechanisms of CIPN.

Cisplatin’s effect on both healthy and cancer cells begins with its cellular uptake. Uptake and accumulation of systemically-administered cisplatin and its metabolites in the DRG allow for platinum-DNA adduct formation and are considered fundamental steps in neurotoxicity development. Preferential accumulation of cisplatin in the DRG results from the presence of an abundant fenestrated capillary network and the absence of the blood-brain barrier [30]. Together, these characteristics of the peripheral nervous system allow for easy access to sensory neurons by exogenous toxins.

Various transport mechanisms have been identified that might allow for uptake of cisplatin into DRG neurons. Two different types of neuronal membrane transporters: volume-regulated anion channel (VRAC), organic cation transporter-2 (OCT2), and copper transporter-1 (CTR1) have been shown to be particularly relevant, and their overexpression in neurons could contribute to the development or exacerbation of neurotoxicity. VRAC mediates anion and osmolyte fluxes to account for cell swelling and changes in tonicity and ionic strength within the cell [33]. It is composed of LRRC8 family members that assemble to form the channel. LRRC8A, an obligatory subunit of VRAC [34], has been shown to play a key role in cisplatin uptake in human embryonic kidney cells as demonstrated by a 70% reduction in cisplatin accumulation following disruption of LRRC8A [35]. Interestingly, DRG neurons have also been shown to express LRRC8A-encoding mRNA, and VRAC currents are inducible within the DRG [36]. Although the specific role VRAC, and specifically the LRRC8A subunit, play in cisplatin-mediated neurotoxicity has not yet been studied, literature suggests a dynamic relationship might exist. Similarly, OCT2 has been extensively studied in the uptake of cisplatin by renal proximal tubular cells leading to cisplatin-induced nephrotoxicity [37,38,39]. OCT2 is also highly expressed in the DRG. Neurons overexpressing mouse OCT2 and human OCT2 have demonstrated a 16- to 35-fold increase in the cellular oxaliplatin accumulation, resulting in a significant increase in DNA platination products and neurotoxicity [40]. Moreover, genetic and pharmacological inhibition of OCT2 has been shown to protect rats from oxaliplatin-induced peripheral neuropathy [41]. Finally, CTR1 is the primary copper influx transporter for cisplatin and has been localized in the DRG of normal rats as well as rats treated with cisplatin [42]. In vivo studies have demonstrated the CTR1-dependent uptake of cisplatin into DRG neurons and the resulting neuronal atrophy [43].

Once intracellular, cisplatin binds to neuronal nuclear and mitochondrial DNA with high affinity [44]. Cisplatin’s antineoplastic effects are achieved through the formation of platination products with nuclear DNA in a highly conserved manner. 1,2-intrastrand d (GpG) (between adjacent guanine bases on the same DNA strand) and 1,2-intrastrand d (ApG) (between adenine and adjacent guanine bases on the same DNA strand) crosslinks are the most common cisplatin-induced adducts [45,46,47]. Unless these DNA-base crosslinks are repaired, they distort DNA’s helical conformation, interrupting replication and transcription. Due to their relatively large size, high metabolic requirements, and long axons, DRG neurons need a high level of active transcription to sustain their physiological processes. In damaged DRG neurons, signaling pathways are eventually induced by the stalling of replication forks and/or RNA polymerases and lead to cell cycle arrest, senescence, or cell death [5,45,46,47]. Importantly, the abundance of adducts correlates to neurotoxicity and has been shown to be three times higher following cisplatin treatment compared to equimolar oxaliplatin doses. This is congruent with in vitro studies demonstrating that cisplatin causes significantly more neuronal cell death than oxaliplatin [17]. In vivo and in vitro studies have demonstrated that cisplatin induces several apoptotic events in neuronal cells, including Bcl-2 suppression [48], activation of p53 [49], Bax translocation, mitochondrial cytochrome c release, and caspase-3 and caspase-9 activation [30]. While these effects of cisplatin on cancer cells are desired for cancer treatment, the same process needs to be avoided in normal tissue to prevent treatment toxicity.

Although cisplatin-induced DNA damage is most widely studied in nuclear DNA, mitochondrial DNA (mtDNA) is not always spared. The first description of mitochondrial dysfunction in DRG neurons as a potential mechanism for cisplatin’s neurotoxicity was published less than 10 years ago. Those studies illustrated that cisplatin binds directly to mitochondrial DNA with a similar affinity as nuclear DNA. Moreover, these cisplatin-mtDNA adducts inhibited mitochondrial transcription and resulted in mitochondrial degradation and vacuolization [50]. Identification of NER proteins in recent years have also raised the question of whether mitochondria are able to undergo DNA repair through NER. CSA, CSB, and PARP1—key NER participants—are imported into the mitochondria in response to oxidative stress and bind to mtDNA, suggesting a possible dynamic role of NER in mitochondrial DNA repair [51].

## 3. Nucleotide Excision Repair

The ability of DRG neurons to repair their DNA after adduct formation is an important determinant of neurotoxicity severity. Without effective DNA repair, chronic cisplatin treatment results in accumulation of DNA-platinum adducts in DRG neurons. Not surprisingly, this accumulation of DNA lesions induces early neurophysiological changes that lead to an increase in neuronal cell death [30]. NER is a major cellular pathway through which cisplatin-induced DNA intrastrand crosslinks are resolved. NER in mammalian cells requires the coordination of major protein groups and can be divided into two subpathways: global-genome NER (GG-NER) and transcription-coupled NER (TC-NER). TC-NER and GG-NER differ in the protein complexes used in the initial recognition of DNA damage. GG-NER, which promotes genomic stability and prevents mutagenesis, requires xeroderma pigmentosum group C, specifically XPC-RAD23B, and DNA damage-binding complexes to survey the genome and recognize helix-distorting DNA crosslinks. TC-NER is initiated by the stalling of RNA polymerase at DNA lesions and signals Cockayne syndrome (CS) proteins CSA and CSB to bind to lesions in the DNA, remove transcription-blocking lesions, and restore transcription. Not surprisingly, TC-NER defects are responsible for multiple genetic disorders whose symptoms include photosensitivity; intellectual, developmental, and physical disability; and the progeria-like features of CS. Both GG-NER and TC-NER rely on XPA to bind to altered nucleotides in ssDNA and facilitate DNA damage verification by the TFIIH complex, thereby launching the NER process [52,53,54,55].

## 4. Proteins Involved in NER-Mediated Modulation of CIPN

### 4.1. APE-1

The primary cellular apurinic/apyrimidinic (AP) endonuclease, human apurinic/apyrimidinic endonuclease 1 (APE1), possesses two major functions—the removal and repair of numerous DNA lesions and redox activation of numerous transcription factors, including Egr-1, NF-κB, p53, and HIF-1α [56]. APE1′s essential role in base excision repair (BER) is the best-characterized mechanism of its DNA repair properties [57] and has demonstrated neuroprotective effects against cisplatin-induced toxicity in isolated sensory neurons [58]. Recent studies have also shown that APE1-mediated cytoprotection of sensory neurons is dependent upon the NER pathway to repair cisplatin-induced DNA damage. Reduction of APE1 expression was shown to inhibit repair of cisplatin-induced DNA damage as demonstrated by a significant decrease in removal of platinum adducts, a process largely mediated by NER. This effect was reversed in add-back experiments, which introduced wtAPE1 using lentivirus and resulted in overexpression of the molecule. Platinum adduct removal was determined to result from APE1-mediated DNA repair, not its redox signaling function, by selectively mutating the molecular region responsible for redox activity. Interestingly, APE1 reduction was shown to alter the expression of RPA70 and XPA, known NER proteins, therefore demonstrating a link between BER and NER. Specifically, knockdown of APE1 led to a decrease in RPA70 and an increase in XPA expression [59]. The significance of this association and applicability to clinical practice remain to be determined.

### 4.2. DNA Polymerase Kappa

While not yet assessed in vivo for symptoms to clearly define a role in CIPN development, DNA polymerase kappa (Pol κ) has been identified as a key regulator of NER in cisplatin-induced DNA damage in DRG neurons [60]. Pol κ, a member of the Y-family of DNA polymerases and the most abundant DNA polymerase in the DRG, has been implicated in repair of various oxidative lesions as well as bulky lesions that are typically NER substrates [61,62,63,64,65]. The study demonstrated cisplatin-induced upregulation of Pol κ in cultured DRG neurons while levels of other DNA polymerases remained stable despite the same genotoxic insult. Cisplatin exposure was also found to induce nuclear Pol κ immunoreactivity in the DRG, and Pol κ depletion resulted in diminished DNA repair synthesis and exacerbation of cisplatin-induced transcriptional suppression [60]. Additional studies assessing the potential role of Pol κ in CIPN using behavioral tests could identify another target for CIPN prevention and treatment.

### 4.3. XPA

Both TC-NER and GG-NER require a key scaffold protein known as XPA to initiate DNA repair. XPA is recruited to a site of DNA damage by the TFIIH complex that is responsible for the creation of the NER bubble and unwinding double-stranded DNA around the damaged nucleotide. Once XPA binds to the repair bubble, it is able to assemble and position additional proteins involved in NER, including XPC, damaged DNA-binding protein 2 (DDB2, also named XPE), XPF/ERCC1, replication protein A (RPA), and proliferating cell nuclear antigen (PCNA) [66]. XPA-deficient mice developed a four-fold increase in DRG DNA adducts following cisplatin treatment compared to wildtype mice. Moreover, XPA-deficient mice exhibited earlier onset of neurophysiological alterations with an inverse correlation between the magnitude of electrophysiological changes and the level of accumulated DNA adducts in DRG neurons [52]. However, XPA also plays an integral role in repair of cisplatin-induced DNA lesions in tumor cells and is required for oncologic control. Patients with metastatic testicular tumors have shown improved prognosis when their tumors have low XPA levels. These findings have also been shown in germ cell tumor patients in which low XPA gene expression conferred significantly better overall survival than patients with high XPA expression [67]. Therefore, selective targeting of XPA in neurons would be imperative to avoid the detrimental effects on oncologic control from systemic XPA deficiency.

### 4.4. PARP-1

Poly(ADP-ribose) polymerase (PARP) has gained much attention due to the recent development of PARP inhibitors and their use in combination with platinum-based cancer treatment regimens [68]. PARP-1 is a chromatin-associated nuclear protein that has been implicated in numerous and varied biological processes including DNA damage protection, DNA repair, transcription regulation, and chromatin remodeling [69]. PARP-1 involvement in single strand break repair and base excision repair is well-established; however, the protein also contributes to NER through interactions with NER proteins. Ultraviolet (UV) radiation exposure was shown to promote an association between PARP-1 and XPA, the key scaffold protein described above. Pharmacologic inhibition of PARP decreased this association in both human keratinocyte extracts in vitro as well as the association between XPA and chromatin-bound PARP-1. Furthermore, PARP inhibition resulted in a reduction of UV radiation-induced XPA binding to chromatin, illustrating a role for PARP-1 in NER through an interaction with XPA [70]. PARP-1 promotion of NER has also been linked to DDB2 stabilization. Enzymatic activity of PARP-1 on DDB2 positively affected the retention time of DDB2 on UV-damaged chromatin, where it is necessary for activation of NER. PARP activity was also necessary to promote stability of DDB2 and effective binding to chromatin [71]. Moreover, PARP-1 has been shown to enhance the interaction between XPC and DDB2 to promote DNA lesion recognition by XPC and initiation of NER [72]. While not yet studied in cisplatin-induced peripheral neuropathy, in vitro studies have shown that PARP is required for repair of cisplatin-DNA adducts in cancer cells [73]. Given the impact of PARP inhibition on cancer control, a major challenge of targeting PARP-1 in treatment of CIPN would be balancing toxicity benefits with oncologic control.

### 4.5. SIRT2

**Figure 1:**
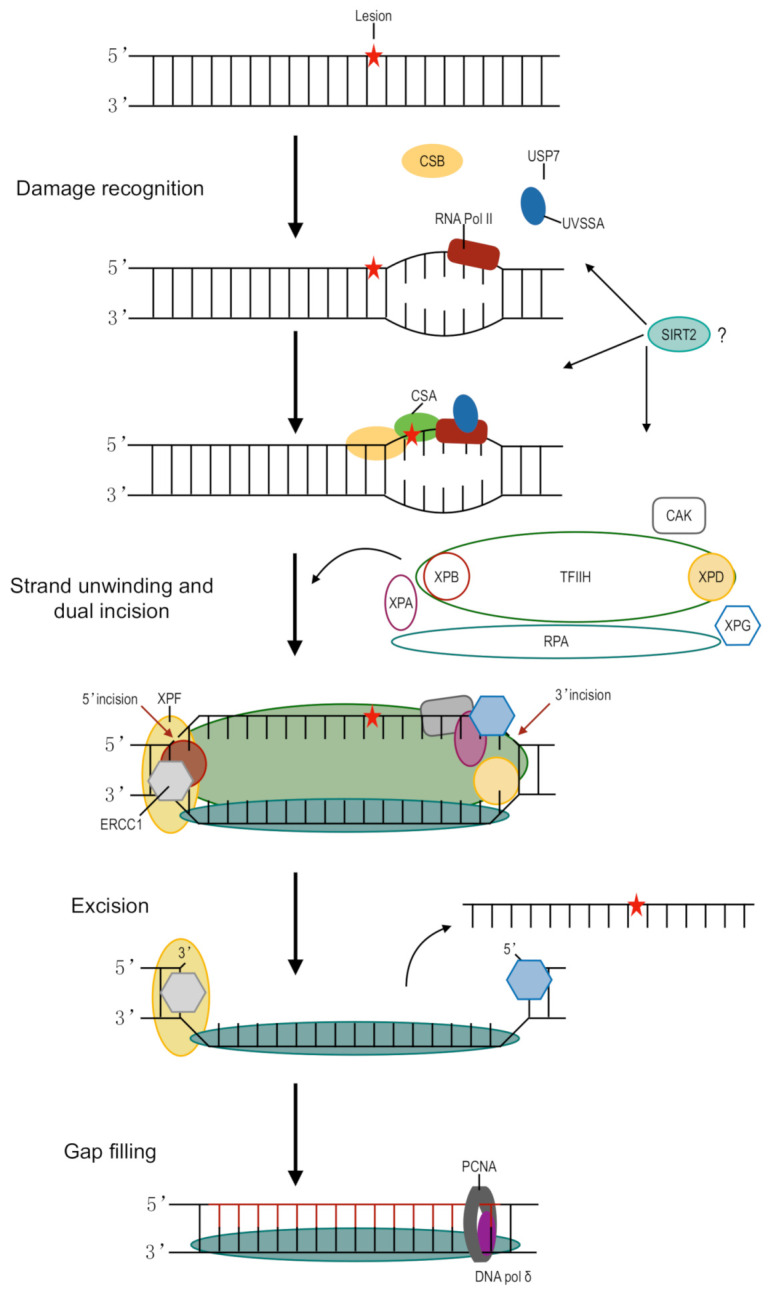
Schematic representation of transcription-coupled nucleotide excision repair. The DNA lesion, as indicated by the red star, stalls transcription by RNA polymerase II (RNA pol II). UVSSA and USP7 stabilize and form a complex with CSB. CSA binds to CSB, likely resulting in backward translocation of RNA pol II and allowing room for NER machinery. TFIIH binds XPG, a structure-specific endonuclease. The multiprotein complex unwinds DNA surrounding the lesion through helicase activity. XPD and XPA act to verify the presence of chemically modified nucleotides in the site of damage. XPF-ERCC1 is directed to the damage by RPA and makes a 5′ incision while XPG is responsible for the 3′ incision. PCNA is loaded onto XPF-ERCC1 and recruits DNA pol δ to fill the gap. SIRT2 promotes TC-NER in DRG, although the exact mechanism is not yet known.

A member of the sirtuin family of NAD^+^-dependent deacetylases, SIRT2, is involved in multiple biological processes including longevity, lipid and glucose homeostasis, tumor suppression, and neurodegenerative disorders. SIRT2 is a nuclear/cytoplasmic protein [74,75], and its subcellular localization and expression level are mediated by stimuli such as diet, oxidative stress, and progression through the cell cycle. Activation of SIRT2 by resveratrol [76] or nicotinamide riboside, a naturally occurring vitamin precursor of NAD+ [77], has been reported to alleviate diabetic neuropathic pain in animal models [78,79]. Previous work from our laboratory demonstrated that SIRT2 protects mice against cisplatin-induced peripheral neuropathy. Cisplatin was shown to induce accumulation of SIRT2 in the nuclei of DRG sensory neurons, and SIRT2 was shown to prevent neuronal cell death in SIRT2-knockin mice. Mechanistically, SIRT2-mediated protection of neurons from cisplatin cytotoxicity was achieved through the promotion of TC-NER of cisplatin-induced DNA crosslinks. Importantly, pharmacologic inhibition of NER with spironolactone abolished SIRT2-mediated TC-NER activity in differentiated neuronal cells and neuroprotection from cisplatin-induced cytotoxicity and CIPN. It is possible that the key players of TC-NER including RPA, CSB, XPA, and XPD (Figure 1) could be directly regulated by SIRT2 through deacetylation or could be indirectly regulated through changes in their expression level due to deacetylation of transcription factors. However, the proteins that act as critical targets of SIRT2 deacetylation in the regulation of NER efficiency and protection of neurons from cisplatin cytotoxicity are not yet known.

## 5. Prevention and Treatment: Current Status and Future Perspectives

Given the frequency, severity, and potential irreversibility of CIPN, extensive efforts have been made to develop preventive and therapeutic strategies for the debilitating toxicity. Nevertheless, CIPN remains unpreventable despite testing numerous preventative therapies. Several clinical trials have investigated potentially neuroprotective agents, yet only duloxetine is moderately recommended by the American Society for Clinical Oncology (ASCO) and no therapies are strongly recommended [80]. Although venlafaxine has a similar mechanism of action to duloxetine, a pilot randomized, placebo-controlled, double-blinded phase III trial (ClinicalTrials.gov: NCT01611155) testing its use in oxaliplatin neurotoxicity prevention in patients with colon cancer did not support the clinical use of venlafaxine to prevent peripheral neuropathy, nor did it support continuation to a full phase III trial [81], see Table 1. Assessment of pregabalin in a phase III randomized, double-blind, placebo-controlled clinical trial (ClinicalTrials.gov: NCT01450163) was similarly unsuccessful. The trial evaluated pregabalin’s efficacy and safety in oxaliplatin prevention and treatment. It was found to be safe but ineffective [82]. Furthermore, trials assessing goshajinkigan, oral vitamin B, and oral alpha-lipoic acid have also failed to identify effective pharmacologic strategies [83,84,85].

For patients who develop CIPN, treatment options are also limited. Current pharmacotherapeutics targeting pain symptoms include analgesics, anticonvulsants, antidepressants, opioids, and serotonin-noradrenalin reuptake inhibitors (SNRIs). Of these, however, duloxetine remains the only one with enough evidence to receive clinical recommendation by ASCO for the treatment of CIPN [80]. Unfortunately, the pain relief seen with duloxetine use is modest and much less robust than desired [93]. Specifically, 59% of patients reported some reduction in pain over the five-week time period with a mean 1.06-point decrease in average pain as assessed by the Brief Pain Inventory-Short Form. As with any drug, duloxetine carries with it the chance of side effects. Most commonly, patients report increased nausea, abdominal pain, fatigue, and headache; however, severe adverse reactions have also been reported [94]. It should also be noted that duloxetine, as well as all SNRIs, are included in the Beers criteria of drugs to avoid in older adults, the population which makes up a large portion of cancer patients.

Despite the challenges faced to find effective strategies for prevention and treatment of CIPN, research continues with this aim. Potential pharmacotherapies include calmangafodipir, L-carnosine, and metformin. Calmangafodipir is being tested in two phase III, placebo-controlled clinical trials (ClinicalTrials.gov: NCT04034355 and NCT03654729) after showing positive results in a placebo-controlled phase II trial of patients receiving oxaliplatin for colorectal cancer [86]. Results of the phase III trials have not yet been reported. L-carnosine was assessed in a pilot randomized controlled trial investigating its efficacy in the prevention of oxaliplatin-induced peripheral neuropathy. Reported results were remarkably positive [87]; however, no placebo was used, and it was not double-blinded. Neuropathy severity was also judged by clinicians as opposed to patient-reported outcomes. Therefore, L-carnosine shows promise for CIPN prevention, but requires additional data. Metformin was also recently evaluated for neuroprotective effects against oxaliplatin-induced neuropathy in a small randomized controlled trial of patients with stage III colorectal cancer. After 12 cycles of FOLFOX-4, significantly reduced grade 2–3 neuropathy and improved patient-reported symptoms were reported [88]. Thus, metformin also shows promise, but requires additional studies with larger sample sizes to evaluate its potential in CIPN prevention.

Nonpharmacologic approaches include exercise, scrambler therapy, and acupuncture. Exercise has been evaluated in multiple randomized controlled trials as a prevention strategy against CIPN. The types of chemotherapy received varied between patients, but those enrolling in standardized aerobic and resistance exercise programs demonstrated a significant reduction in peripheral neuropathy symptoms compared to those not receiving intervention [89,95,96]. Results from these studies have prompted ongoing trials investigating the utility of exercise in CIPN prevention (ClinicalTrials.gov: NCT03858153). Scrambler therapy (ST), an electrocutaneous treatment, was tested in two phase II RCTs. The smaller trial (N = 33) found no significant differences between the groups receiving ST versus the sham group [90]. The other trial compared ST to transcutaneous electrical nerve stimulation (TENS) and showed more improvement in neuropathy symptoms, pain, and quality of life in patients receiving ST [91]. Given the small sample sizes and inconclusive results, ASCO is not currently able to recommend ST outside of a clinical trial. Finally, acupuncture has been evaluated in multiple trials. One example is a randomized assessor-only-blinded controlled trial in 87 patients receiving unspecified chemotherapy that found significant reduction of pain assessed by the Brief Pain Inventory and improvement in neurologic assessment, quality of life, and symptom distress. Many of the improvements seen were long-lasting, including physical and functional well-being at a 20-week evaluation [92]. Another smaller trial of patients with cancer and CIPN found significant improvement in physical and function areas of the European Organization for Research and Treatment of Cancer (EORTC) Quality of Life Questionnaire-Core 30 after 10 sessions of acupuncture [97].

Given NER’s key role in repair of cisplatin-induced DNA crosslinks, it is reasonable to investigate NER-targeted prevention and treatment strategies against CIPN. NER could be targeted through the newly discovered mediator, SIRT2, which has also been linked to many of the strategies currently under clinical investigation for CIPN prevention and treatment. Metformin [98], exercise [99,100], and acupuncture [101] have all been shown to mediate various physiologic functions through SIRT2. Importantly, pharmacologic approaches for SIRT2 activation include both resveratrol and nicotinamide riboside [102] and have been used in numerous clinical studies.

## 6. Conclusions

With increasing survival rates of cancer patients [103], CIPN has become a significant, debilitating cause of decreased quality of life in patients receiving chemotherapy. Although extensive investigation into the mechanisms underlying CIPN and potential strategies for its prevention and treatment have provided deeper understanding into the complex topic, effective clinical strategies remain elusive. The mechanism of action of both anticancer functions and toxicity relies heavily on DNA damage; however, other pathways likely play supporting roles. NER is integral to the repair of DNA damage induced by platinum agents and thus, targeting of its mediators could result in discovery of strategies for CIPN prevention and treatment. As interest in chemotherapy toxicity and a focus on improving quality of life of cancer patients continues to grow, finding effective pharmacologic and nonpharmacologic approaches to address CIPN remains possible.

## Figures and Tables

**Table 1:** Prevention and treatment of CIPN in prospective randomized clinical trials.

Intervention	Agent	Sample Size	Outcome Measured	Results	Reference
Venlafaxine (V)	Oxaliplatin	50 V: 25 PL: 25	EORTC QLQ-CIPN20 sensory subscale	No significant difference in CIPN20 score over 12 cycles of oxaliplatin between V and PL *p* = 0.55	Zimmerman 2016 [81]
Pregabalin (PG)	Oxaliplatin	143 PG: 78 PL: 65	Avg. pain on BPI at 6 months	PG: 1.03 PL: 0.85 *p* = n.s.	de Andrade 2017 [82]
Vitamin B (Vit. B)	Taxanes, oxaliplatin, or vincristine	47 Vit. B: 27 PL: 22	Total neuropathy score (TNS)	No significant difference in TNS at the 12, 24, and 36 weeks *p* = n.s.	Schloss 2017 [84]
Oral alpha-lipoic acid (ALA)	Cisplatin, oxaliplatin	243 ALA: 122 PL: 121	FACT/GOG-Ntx score	No significant difference in FACT/GOG-Ntx scores at 24 weeks *p* = n.s.	Guo 2014 [85]
Calmangafodipir (Cal)	Oxaliplatin (FOLFOX-6)	173 Cal 2 μmol/kg = 57; 5 μmol/kg = 45, 10 μmol/kg = 11 PL = 60	NCI-CTCAE; Leonard Scale Questionnaire; NCI-Sanofi; cold allodynia test	Trend toward decreased physician graded neurotoxicity *p* = 0.16 Decreased patient-reported symptoms *p* < 0.01	Glimelius 2018 [86]
L-Carnosine (LC)	Oxaliplatin (FOLFOX-6)	65 LC: 34 No LC: 31	NCI-CTCAE at 3 months	Grade 1 toxicity: LC: 56.7%, No LC: 35.5% Grade 2: LC: 3.3%, No LC: 61.3%, No toxicity: LC:40%, No LC: 3.2%, *p* < 0.05	Yehia 2019 [87]
Metformin (Met)	Oxaliplatin (FOLFOX-4)	40 Met: 20 No Met: 20	NCI-CTCAE	Grade 2-3 toxicity: Met: 60%, No Met: 95% *p* = 0.009	El-fatatry 2018 [88]
Exercise (E)	Taxane-, platinum-, or Vinca alkaloid-based	355 E: 155 C: 185	Patients reported grading of CIPN symptoms	Decreased temperature sensitivity *p* = 0.045 Decreased sensory symptoms *p* = 0.061	Kleckner 2018 [89]
Scrambler therapy (ST)	Not specified	35 ST: 17 Sham: 18	Numeric Rating Scale	Avg. pain at 28 days: ST: 5.79, Sham: 5.44 *p* = 0.606	Smith 2020 [90]
ST vs. transcutaneous electrical nerve stimulation (TENS)	“Neurotoxic chemotherapy”	46 ST: 24 TENS: 22	Proportion of pts who achieve at least 50% reduction in pain or tingling after 2 weeks	ST: 40% TENS: 20% *p* = 0.12	Loprinzi 2020 [91]
Acupuncture (AC)	Taxanes, bortezomib, capecitabine, or platinum-based chemotherapy	87 AC: 44 C: 43	BPI	Worst pain at baseline: AC: 2.1, C: 1.3; 8 weeks: AC: 1.0, C: 1.7, *p* = 0.008; 20 weeks: AC:1.8, C: 2.3, *p* = 0.49	Molassiotis 2019 [92]

PL: placebo; C: control; EORTC QLQ-CIPN20: European Organization for Research and Treatment of Cancer Quality of Life Questionnaire-CIPN 20-item scale; BPI: Brief Pain Inventory; FACT/GOG-Ntx: Functional Assessment of Cancer Therapy/Gynecology Oncology Group-Neurotoxicity; NCI-CTCAE: National Cancer Institute Common Terminology Criteria for Adverse Events; Pts: patients.

## Abbreviations

CIPN	chemotherapy-induced peripheral neuropathy
NER	nucleotide excision repair
DSB	double-strand break
DRG	dorsal root ganglia
